# Influenza Vaccination Coverage Among Health Care Personnel — United States, 2017–18 Influenza Season

**DOI:** 10.15585/mmwr.mm6738a2

**Published:** 2018-09-28

**Authors:** Carla L. Black, Xin Yue, Sarah W. Ball, Rebecca V. Fink, Marie A. de Perio, A. Scott Laney, Walter W. Williams, Samuel B. Graitcer, Amy Parker Fiebelkorn, Peng-Jun Lu, Rebecca Devlin

**Affiliations:** ^1^Immunization Services Division, National Center for Immunization and Respiratory Diseases, CDC; ^2^Leidos, Reston, Virginia; ^3^Abt Associates Inc., Cambridge, Massachusetts; ^4^Division of Surveillance, Hazard Evaluations, and Field Studies, National Institute for Occupational Safety and Health, CDC; ^5^Division of Respiratory Health, National Institute for Occupational Safety and Health, CDC.

The Advisory Committee on Immunization Practices (ACIP) recommends that all health care personnel receive an annual influenza vaccination to reduce influenza-related morbidity and mortality among health care personnel and their patients and to reduce absenteeism among health care personnel (*1*–*4*). CDC conducted an opt-in Internet panel survey of 2,265 U.S. health care personnel to estimate influenza vaccination coverage among these persons during the 2017–18 influenza season. Overall, 78.4% of health care personnel reported receiving influenza vaccination during the 2017–18 season, similar to reported coverage in the previous four influenza seasons (*5*). As in previous seasons, coverage was highest among personnel who were required by their employer to be vaccinated (94.8%) and lowest among those working in settings where vaccination was not required, promoted, or offered on-site (47.6%). Health care personnel working in long-term care settings, the majority of whom work as assistants or aides, have lower influenza vaccination coverage than do health care personnel working in all other health care settings, which puts the elderly in long-term settings at increased risk for severe complications for influenza. Implementing workplace strategies shown to improve vaccination coverage among health care personnel, including vaccination requirements and active promotion of on-site vaccinations at no cost, can help ensure health care personnel and patients are protected against influenza (*6*). CDC’s long-term care web-based toolkit* provides resources, strategies, and educational materials for increasing influenza vaccination among health care personnel in long-term care settings.

An Internet panel survey of health care personnel was conducted for CDC during March 27–April 17, 2018, to provide estimates of influenza vaccination coverage among health care personnel during the 2017–18 influenza season. Similar surveys have been conducted since the 2010–11 influenza season, and survey methodology has been described previously (*7*). Respondents were recruited from two preexisting national opt-in Internet sources: Medscape, a medical website managed by WebMD Health Professional Network,^†^ and general population Internet panels operated by Survey Sampling International (SSI).^§^ Responses were weighted to the distribution of the U.S. population of health care personnel by occupation, age, sex, race/ethnicity, work setting, and Census region.^¶^ Because the study sample was based on health care personnel from opt-in Internet panels rather than probability samples, statistical tests were not conducted.** A change was considered an increase or decrease when there was at least a 5 percentage-point difference between estimates; estimates with smaller differences were considered similar.

Among the 2,382 persons who started the survey from either source (Medscape or SSI) and had eligible responses to the screening questions, 2,310 (97.0%) completed the survey.^††^ Forty-three respondents with completed surveys who reported working in “other health care settings” were excluded because examination of their other survey responses indicated that they were either unlikely to have contact with patients or unlikely to have worked in one of the health care settings of interest for this analysis; two additional respondents were excluded because their work locations were outside of the United States. The final analytic sample included 2,265 health care personnel.

Overall, 78.4% of health care personnel reported having received an influenza vaccination during the 2017–18 season, a 15 percentage-point increase since the 2010–11 season but similar to coverage in the previous four seasons (75.2%–78.6%) (Figure 1) (Figure 2). Vaccination coverage in the 2017–18 season was similar to that in the 2016–17 season among health care personnel in all work settings (Figure 1) and occupation groups (Figure 2). As in previous seasons, coverage in the 2017–18 season was highest among health care personnel working in hospital settings (91.9%) followed by those working in ambulatory care (75.1%), other clinical settings (74.9%), and long-term care settings (67.4%) (Figure 1). Overall, vaccination coverage in 2017–18 was higher among physicians (96.1%), pharmacists (92.2%), nurses (90.5%), and nurse practitioners and physician assistants (87.8%), and lower among other clinical health care personnel (80.9%), assistants and aides (71.1%), and nonclinical health care personnel (72.8%) (Figure 2).

**FIGURE 1:**
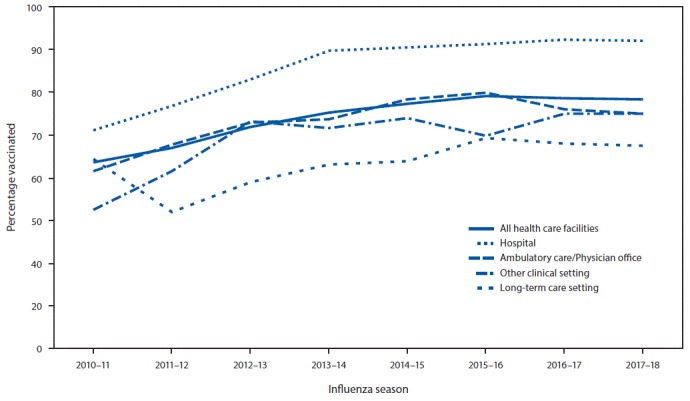
Percentage of health care personnel who received influenza vaccination, by work setting* — Internet panel surveys,^†^ United States, 2010–11 through 2017–18 influenza seasons * Respondents could select more than one work setting. The “ambulatory care/physician office” category includes physician’s office, medical clinic, and other ambulatory care settings. The “other clinical setting” category includes dentist office or dental clinic, pharmacy, laboratory, public health setting, emergency medical services setting, or other setting where clinical care or related services were provided to patients. ^†^ Respondents were recruited from two preexisting national opt-in Internet sources: Medscape, a medical website managed by WebMD Health Professional Network, and general population Internet panels operated by Survey Sampling International.

**FIGURE 2:**
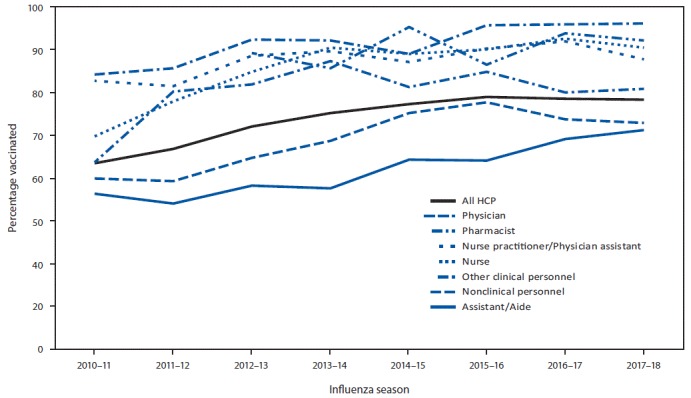
Percentage of health care personnel (HCP) who received influenza vaccination, by occupation* — Internet panel surveys,† United States, 2010–11 through 2017–18 influenza seasons * In the 2010–11 season, dentists were included in the physician category. Before the 2012–13 season, separate data on pharmacists were not collected. Other clinical personnel category includes allied health professionals, technicians, and technologists. Nonclinical personnel category includes administrative support staff members or managers and nonclinical support staff members (e.g., food service workers, laundry workers, janitors, and other housekeeping and maintenance staff members). ^†^ Respondents were recruited from two preexisting national opt-in Internet sources: Medscape, a medical website managed by WebMD Health Professional Network, and general population Internet panels operated by Survey Sampling International.

Vaccination coverage was highest (94.8%) among health care personnel working in settings where vaccination was required (Table). Overall, 44.1% of health care personnel reported a requirement to be vaccinated; those working in hospitals were more likely to report a vaccination requirement (68.3%) than were those working in ambulatory care (39.2%), long-term care (29.6%), or other clinical settings (37.9%) (Table). Among health care personnel whose employers did not have a requirement for vaccination, coverage was higher among those who worked in locations where vaccination was offered at the worksite at no cost for 1 day only (70.4%) or >1 day (76.0%) or who worked in locations where their employer did not provide influenza vaccination on-site at no cost but actively promoted vaccination through other mechanisms^§§^ (75.1%) compared with that among health care personnel working in locations where employers did not have any vaccination-related requirements or provisions (47.6%) (Table). Health care personnel working in hospital settings were less likely to report that their employer did not require, provide, or promote vaccination (2.1%) than were personnel working in ambulatory care, long-term care, and other clinical settings (23.2%, 23.5%, and 26.1%, respectively).

**TABLE:** Percentage of health care personnel* who received influenza vaccination, by employer vaccination requirements, workplace vaccine availability, and work setting — Internet panel surveys,^†^ United States, 2017–18 influenza season

Vaccination requirement and availability/Work setting	No. (weighted %^§^)	Weighted % vaccinated
**Employer vaccination requirement** ^¶^	**921 (44.1)**	**94.8**
Hospital	572 (68.3)	96.6
Ambulatory care/Physician office**	267 (39.2)	91.2
Long-term care	161 (29.6)	89.3
Other clinical setting^††^	200 (37.9)	90.1
**On-site vaccination >1 day^§§^**	**380 (14.3)**	**76.0**
Hospital	97 (14.8)	85.2
Ambulatory care/Physician office**	101 (13.1)	79.1
Long-term care	76 (13.9)	59.4
Other clinical setting^††^	155 (15.4)	76.7
**On-site vaccination 1 day^¶¶^**	**315 (14.6)**	**70.4**
Hospital	62 (11.5)	80.3
Ambulatory care/Physician office**	91 (16.0)	70.1
Long-term care	101 (17.4)	67.4
Other clinical setting^††^	91 (9.4)	67.0
Other vaccination promotion***	**218 (9.6)**	**75.1**
Hospital	20 (3.3)	—^†††^
Ambulatory care/Physician office**	42 (8.4)	74.2
Long-term care	94 (15.6)	70.4
Other clinical setting^††^	76 (11.2)	74.0
**No requirement, on-site vaccination or promotion**	**431 (17.4)**	**47.6**
Hospital	31 (2.1)	39.9
Ambulatory care/Physician office**	120 (23.2)	49.4
Long-term care	148 (23.5)	42.4
Other clinical setting^††^	166 (26.1)	54.9

* Persons who worked in a place where clinical care or related services were provided to patients, or whose work involved face-to-face contact with patients or who were ever in the same room as patients.

^†^ Respondents were recruited from two preexisting national opt-in Internet sources: Medscape, a medical website managed by WebMD Health Professional Network, and general population Internet panels operated by Survey Sampling International.

^§^ Weights were calculated based on each occupation type, by age, sex, race/ethnicity, work setting, and U.S. Census region to represent the U.S. population of health care personnel. Work setting and overall occupation are presented as weighted estimates of the total sample. Where the groups are stratified by work setting, the estimates are presented as weighted estimates of the occupation group subsample of each work setting subgroup.

^¶^ Includes all respondents who indicated that their employer required them to be vaccinated for influenza.

** Ambulatory care (physician’s office, medical clinic, and other ambulatory care setting).

^††^ Dentist office or dental clinic, pharmacy, laboratory, public health setting, health care education setting, emergency medical services setting, or other setting where clinical care or related services was provided to patients.

**^§§^** Employer made influenza vaccination available on-site for >1 day during the influenza season at no cost to employees. Restricted to respondents without an employer requirement for vaccination.

^¶¶^ Employer made influenza vaccination available on-site for 1 day during the influenza season at no cost to employees. Restricted to respondents without an employer requirement for vaccination.

*** Influenza vaccination was promoted among employees through public identification of vaccinated persons, financial incentives, or rewards to individuals or groups of employees, competition between units or care areas, free or subsidized cost of vaccination, personal reminders to be vaccinated, or publicizing of the number or percentage of employees receiving vaccination. Restricted to respondents without an employer requirement for vaccination or on-site vaccination.

^†††^ Vaccination coverage estimate not reliable because the sample size was <30.

## Discussion

The overall influenza vaccination coverage estimate among health care personnel was 78.4% during the 2017–18 influenza season, a 15 percentage-point increase since the 2010–11 season, but similar to coverage during the previous four seasons (*5*). As in past seasons, the highest coverage was associated with workplace vaccination requirements. Reported coverage was consistently higher among health care personnel working in hospital settings than among those working in other settings; health care personnel working in hospital settings were also the most likely to report workplace vaccination requirements. Influenza vaccination coverage was higher among health care personnel with vaccination available at or promoted in their workplace than among those without any type of employer promotion of vaccination; however, coverage achieved through vaccine availability and promotion was still suboptimal in the absence of requirements. Neither vaccination coverage nor prevalence of employer vaccination requirements or promotion differed in the 2017–18 season compared with the previous season (*5*), despite the severity of the 2017–18 influenza season (*8*).

Influenza vaccination coverage among health care personnel working in long-term care settings, the majority of whom work as assistants and aides (*5*,*7*), continues to be consistently lower than that among health care personnel working in all other health care settings. Influenza vaccination among health care personnel in long-term care settings is especially important because influenza vaccine efficacy is generally lowest among the elderly, who are at increased risk for severe disease (*2*). In contrast to health care personnel working in hospitals, a much lower proportion of survey respondents working in long-term care settings reported having a requirement for vaccination, and 23.5% reported that their employer did not require, make available on-site at no cost, or promote vaccination in any way. Implementing workplace vaccination programs that have been successful in increasing coverage in hospital settings, including vaccination requirements, could increase coverage in long-term care and other settings with historically lower vaccination coverage.

The findings in this report are subject to at least three limitations. First, the study used a nonprobability sample of volunteer members of Medscape and SSI Internet panels. Second, vaccination status was self-reported and might be subject to recall bias. Finally, coverage findings from Internet survey panels have differed from population-based estimates from the National Health Interview Survey in past influenza seasons, although trends in coverage were similar across seasons (*9*,*10*).

The highest influenza vaccination coverage among health care personnel continues to be reported in worksites with employer requirements for vaccination. Numerous professional medical associations, including the American Medical Directors Association, the Society for Healthcare Epidemiology of America, the American Hospital Association, the American College of Physicians, the American Nurses Association, and the American Pharmacists Association support mandatory influenza vaccination requirements for health care personnel.^¶¶^ In the absence of vaccination requirements, recommendations found in the Guide to Community Preventive Services, which include actively promoted on-site vaccination at no or low cost, can increase influenza vaccination coverage among health care personnel (*6*), although promotional activities generally do not attain the levels of coverage achieved by vaccination requirements. Long-term care employers can use CDC’s long-term care web-based toolkit, which provides access to resources, strategies, and educational materials for increasing influenza vaccination among health care personnel and reducing influenza-associated morbidity and mortality among patients in long-term care settings.

**Summary**

**What is already known about this topic?**

Annual influenza vaccination is recommended for health care personnel to reduce influenza-related morbidity and mortality.

**What is added by this report?**

Opt-in Internet panel survey-assessed influenza vaccination coverage among health care personnel during the 2017–18 season was 78.4%, similar to the previous four seasons. Employer vaccination requirements and offering/promoting workplace vaccination were associated with higher coverage; coverage was lowest among long-term care setting personnel, who were least likely to report employer vaccination requirements or workplace vaccine availability/promotion.

**What are the implications for public health practice?**

Implementing comprehensive evidence-based worksite intervention strategies is important to ensure health care personnel and patients are protected against influenza. To protect the elderly from severe influenza complications, CDC tools are available for increasing vaccination among long-term care setting personnel.

## Footnotes

* https://www.cdc.gov/flu/toolkit/long-term-care/index.htm.

† Physicians, nurse practitioners, physician assistants, nurses, dentists, pharmacists, allied health professionals, technicians, and technologists were recruited from the current membership roster of Medscape. Additional information on Medscape is available at https://www.medscape.com.

§ Assistants, aides, and nonclinical personnel (such as administrators, clerical support workers, janitors, food service workers, and housekeepers) were recruited from general population Internet panels operated by Survey Sampling International. Additional information on Survey Sampling International and its incentives for online survey participants is available at https://www.surveysampling.com.

¶ Population control totals of U.S. health care personnel by occupation and work setting were obtained from the Bureau of Labor Statistics, U.S. Department of Labor, Occupational Employment Statistics, May 2016 National Industry-Specific Occupational Employment and Wage Estimates (https://www.bls.gov/oes/current/oessrci.htm). Population control totals by other demographic characteristics were obtained from the U.S. Census Bureau, Current Population Survey Monthly Labor Force Data, September 2017 (https://www.bls.gov/cps/data.htm).

** Additional information on obstacles to inference in nonprobability samples is available at: https://www.aapor.org/AAPOR_Main/media/MainSiteFiles/NPS_TF_Report_Final_7_revised_FNL_6_22_13.pdf and https://www.aapor.org/getattachment/Education-Resources/For-Researchers/AAPOR_Guidance_Nonprob_Precision_042216.pdf.aspx. While the estimates reported here have variance, there has been no attempt to quantify the size of the variance.

†† A survey response rate requires specification of the denominator at each stage of sampling. During recruitment of an online opt-in survey sample, such as the Internet panels described in this report, these numbers are not available; therefore, a response rate cannot be calculated. Instead, the survey completion rate is provided.

§§ Employer promoted influenza vaccination among employees through public identification of vaccinated persons, financial incentives or rewards to individual persons or groups of employees, competition between units or care areas, free or subsidized cost of vaccination, personal reminders to be vaccinated, or publicizing of the number or percentage of employees receiving vaccination.

¶¶ Position statements of professional organizations that endorse influenza vaccination requirements for health care personnel can be found at: http://www.immunize.org/honor-roll/influenza-mandates/. The statement of the American Nurses Association can be found at: https://www.nursingworld.org/~49177c/globalassets/docs/ana/executivesummarypositionstatement_immunizations.pdf.
